# Effect of Cerebral Small Vessel Disease Burden on Infarct Growth Rate and Stroke Outcomes in Large Vessel Occlusion Stroke Receiving Endovascular Treatment

**DOI:** 10.3390/biomedicines11113102

**Published:** 2023-11-20

**Authors:** Jong-Hee Sohn, Yejin Kim, Chulho Kim, Joo Hye Sung, Sang-Won Han, Yerim Kim, Soo-Hyun Park, Minwoo Lee, Kyung-Ho Yu, Jae Jun Lee, Sang-Hwa Lee

**Affiliations:** 1 Department of Neurology, Chuncheon Sacred Heart Hospital, Hallym University College of Medicine, 77 Sakju-ro, Chuncheon 24253, Republic of Korea; deepfoci@hallym.or.kr (J.-H.S.); gumdol52@hallym.or.kr (C.K.); centertruth@naver.com (J.H.S.); rabiting0@daum.net (S.-W.H.); 2 Institute of New Frontier Research Team, Hallym University, Chuncheon 24252, Republic of Korea; hiruizhen@naver.com (Y.K.); iloveu59@hallym.or.kr (J.J.L.); 3 Department of Neurology, Kangdong Sacred Heart Hospital, Hallym University College of Medicine, Seoul 05355, Republic of Korea; brainyrk@gmail.com (Y.K.); g2skhome@gmail.com (S.-H.P.); 4 Department of Neurology, Hallym Sacred Heart Hospital, Hallym University College of Medicine, Anyang 14068, Republic of Korea; minwoo.lee.md@gmail.com (M.L.); ykh1030@hallym.or.kr (K.-H.Y.); 5 Department of Anesthesiology and Pain Medicine, Chuncheon Sacred Heart Hospital, Hallym University College of Medicine, Chuncheon 24252, Republic of Korea

**Keywords:** cerebral small vessel disease, infarct growth, large vessel occlusion, stroke progression, prognosis, endovascular treatment

## Abstract

This study aimed to investigate the association between cerebral small vessel disease (CSVD) burden and infarct growth rate (IGR) in patients with large vessel occlusion (LVO) stroke who underwent endovascular treatment (EVT). A retrospective analysis was conducted on a cohort of 495 patients with anterior circulation stroke who received EVT. CSVD burden was assessed using a CSVD score based on neuroimaging features. IGR was calculated from diffusion-weighted imaging (DWI) lesion volumes divided by the time from stroke onset to imaging. Clinical outcomes included stroke progression and functional outcomes at 3 months. Multivariate analyses were performed to assess the relationship between CSVD burden, IGR, and clinical outcomes. The fast IGR group had a higher proportion of high CSVD scores than the slow IGR group (24.4% vs. 0.8%, *p* < 0.001). High CSVD burden was significantly associated with a faster IGR (odds ratio [95% confidence interval], 26.26 [6.26–110.14], *p* < 0.001) after adjusting for confounding factors. High CSVD burden also independently predicted stroke progression and poor functional outcomes. This study highlights a significant relationship between CSVD burden and IGR in LVO stroke patients undergoing EVT. High CSVD burden was associated with faster infarct growth and worse clinical outcomes.

## 1. Introduction

Cerebral small vessel disease (CSVD) represents a group of pathological features affecting intracranial arterioles, venules, and capillaries that can cause lacunes, white matter hyperintensities (WMH), cerebral microbleeds (CMB), and enlarged perivascular spaces (EPVSs) on neuroimaging [1,2]. Recently, emerging evidence has indicated that CSVD burden increased the worse prognosis in acute ischemic stroke patients with endovascular treatment (EVT) [3,4,5,6].

As the current principle of EVT is based on the ischemic core and ischemic penumbra concepts, the subsequent growth of ischemic lesions after large vessel occlusion (LVO) might be of therapeutic interest in reperfusion therapy in acute stroke settings [7,8]. When patients with a rapid infarct growth rate (IGR) had a large ischemic core and fewer penumbral salvage lesions, they had unfavorable outcomes after reperfusion therapy, compared with those with a slow IGR [9,10]. However, the clinical implication of the IGR on diffusion-weighted imaging (DWI) lesions has received little attention. The DEFUSE 3 study demonstrated an extension of the therapeutic window for reperfusion therapy up to 16 h, and a slower IGR may be one of the factors contributing to the extended therapeutic window [11]. Therefore, the evaluation of markers for accelerated infarct growth could have important clinical implications in the era of EVT.

The CSVD might be attributed to cerebral blood flow, perfusion impairment, and blood–brain barrier (BBB) permeability alterations in pathophysiology [12]. Although still controversial, there have also been experimental and clinical studies showing that CSVD is associated with collateral circulation via the vasodilatory response [3,13]. Therefore, we assumed that CSVD burden might affect IGR before EVT in LVO stroke patients. Although several factors could affect IGR after stroke onset, there has been no study evaluating the association between CSVD burden and IGR in LVO stroke [14,15,16].

Using a multicenter registry database, we evaluated the effect of CSVD burden on IGR and stroke outcome in LVO stroke patients treated with EVT.

## 2. Materials and Methods

### 2.1. Study Population

We consecutively registered acute ischemic stroke patients in web-based registry database of four university-affiliated institutions. We included patients with acute ischemic stroke with anterior circulation proximal LVO from August 2015 to May 2023 in this study. For the aim of this study, we excluded the following patients: (1) those with pre-stroke-modified Rankin Scale (mRS) ≥2; (2) those who did not undergo brain magnetic resonance imaging (MRI) at hospitalization; (3) those with Alberta Stroke Program Early CT score (ASPECTS) < 6; (4) those with an interval from stroke onset to groin puncture over 16 h; (5) those with poor collateral status via multiphasic CT angiography (mCTA); (6) those with unwitnessed stroke onset with symptom discovery over 1 h from last known period of wellness [16].

### 2.2. Clinical Data and Definition of Parameters

We obtained demographic, clinical, laboratory, and outcome data directly from the web-based registry databases of four institutions. Stroke-related characteristics, including initial stroke severity using the National Institutes of Health Stroke Scale (NIHSS) score, and stroke subtype based on the Trial of Org 10,172 in Acute Stroke Treatment (TOAST) classification, were also obtained [17]. Stroke treatment status including preceding intravenous thrombolysis and intervals from stroke onset to groin puncture time were collected. The core laboratory data were also collected. As outcome data, early neurological deterioration and its etiologies and the modified Rankin Scale at 3 months after stroke were consecutively collected in the registry database.

### 2.3. Assessment of CSVD Burden

We identified CSVD burden according to the STandards for ReportIng Vascular changes on nEuroimaging (STRIVE) criteria, as an MRI feature of CSVD [1,18]. These imaging markers of CSVD were assessed by two expert vascular neurologists (M Lee and S-H Lee) in a double-blinded manner (interclass correlation coefficient [ICC] = 0.90, *p* < 0.001). The SVD burden score was estimated according to previous study by summing the presence of each of the 4 MRI features of CSVD: 1 point for the presence of ≥1 lacune(s), 1 point for the presence of ≥1 CMB(s), 1 point for the presence of moderate to severe EPVS (score 2–4), 1 point for periventricular WMHs Fazekas 3 and/or deep WMH Fazekas 2–3. Hence, the total CSVD score ranges from 0 to 4 [19]. We categorized CSVD score as low (0,1,2) and high CSVD (3 and 4).

### 2.4. Definitions of Imaging Biomarkers

All enrolled participants performed mCTA and brain MRI using a 3T whole-body MRI system. IGR for all patients was defined using the initial infarct volume on DWI divided by the time interval from stroke onset to initial MRI [10]. Infarct volume on DWI was calculated using Processing, Analyzing, Visualization (MIPAV version 7.4.0, NIH, Bethesda, MD, USA) software through the semiautomated volumetric analysis method [20]. We also assessed the ASPECTS using initial brain non-contrast computed tomography and collateral status using mCTA. The leptomeningeal collateral status was assessed according to the imaging protocols of the Calgary Stroke Program and classified as good, intermediate, or poor collateral status [21]. The successful reperfusion after EVT was defined as modified Thrombolysis in Cerebral infarction scale (TICI) grade 2b or 3. Since each center belongs to the same foundation, it has the advantage of using a consistent imaging protocol among hospitals. All the imaging biomarkers were evaluated by two expert vascular neurologists (M Lee and S-H Lee) in a double-blinded manner (ICC’s > 0.88 *p*’s < 0.001).

### 2.5. Outcome Measures

The primary outcome measure was IGR and was determined using the initial DWI volume divided by the time between stroke onset and the MRI. This approach assumed a DWI volume of zero prior to stroke onset [10]. We defined fast IGR as a higher value of optimal IGR for predicting stroke progression. We established the optimal IGR cutoff for predicting stroke progression using the receiver operating characteristic (ROC) curve. The secondary outcome measures were early stroke progression and poor functional outcome at 3 months. Early stroke progression was defined as an increase of at least 1 point in motor power or a deterioration of ≥2 points in the total NIHSS score within 72 h of hospitalization as compared with the initial NIHSS score [22]. The poor functional outcome was defined as mRS > 2 at 3 months.

### 2.6. Statistical Analysis

For descriptive statistics, categorical variables are presented as the percentage of subjects, while for continuous variables, they are presented as mean ± SD or median (interquartile range, IQR). Appropriate tests were used to compare differences between groups, such as Pearson’s chi-square test or Fisher’s exact test for categorical variables, and Student’s *t*-test or Mann–Whitney U test for continuous variables.

This study compared baseline, clinical, and imaging variables between the slow and fast IGR groups. We established the optimal IGR cutoff for predicting stroke progression using the receiver operating characteristic curve. Logistic regression analysis was used to investigate the independent effect of high CSVD on fast IGR. Variables with *p*-values less than 0.1 in the univariate comparisons, as well as clinically plausible factors, such as age, sex, stroke etiology, and initial NIHSS, were included as covariates in the multivariate analysis.

As a sensitivity analysis, we conducted a logistic regression analysis to assess the impact of each CSVD marker (deep WMM Fazekas 2–3, periventricular WMH 3, EPVS, MB, and lacune) on IGR. All statistical analyses were performed using IBM SPSS v21.0 (IBM Corporation, Armonk, NY, USA) and R v4.0.3 (R Foundation for Statistical Computing, Vienna, Austria).

## 3. Results

**Figure 1:**
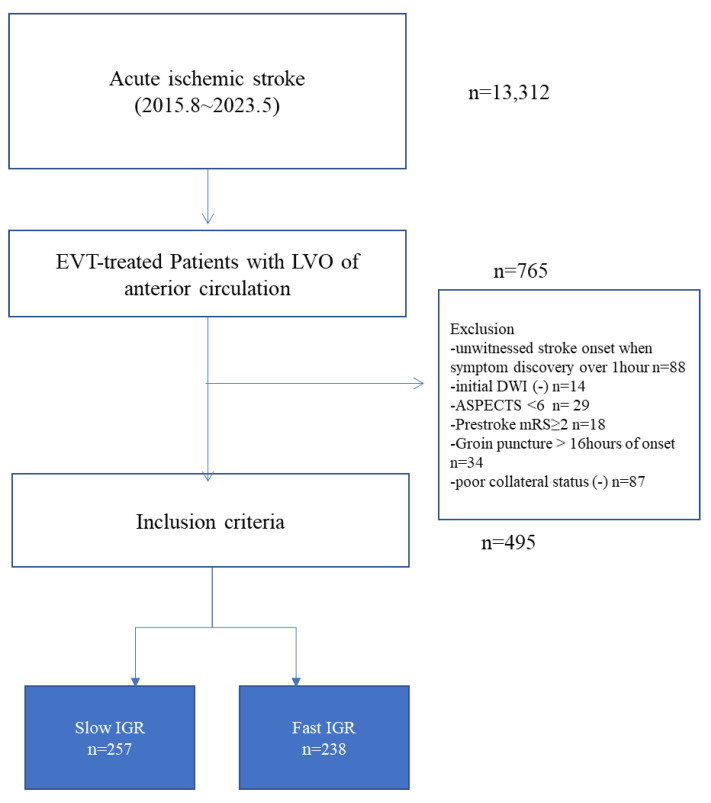
Flow chart of study. Abbreviations: EVT, endovascular treatment; LVO, large vessel occlusion; DWI, diffusion-weighted image; ASPECT, Alberta Stroke Program Early CT score; mRS, modified Rankin Scale; IGR, infarct growth rate.

Of the 13,312 consecutive acute ischemic stroke patients, 765 with anterior circulation stroke underwent EVT at four different institutions. Out of these, 495 patients fulfilled the criteria for inclusion (Figure 1). Their mean age was 69.8 ± 12.9 years, 58.0% were male, and they had a median initial NIHSS score of 15 (IQR 10–18) and median IGR of 7.4 (IQR 1.8–31.6). The mean time from stroke onset to arrival was 3.5 ± 4.9 h, while the time from stroke onset to MRI was 3.9 ± 4.9 h. Based on the ROC curve results, the optimal IGR for predicting stroke progression was found to be 8.0 mL/h. Of 495 patients, 238 patients (48.1%) were classified as the fast IGR group (IGR ≥ 8.0 mL/h) and 257 (51.9%) as the slow IGR group (IGR < 8.0 mL/h).

The fast IGR group showed more severe stroke symptoms, arrived for imaging and treatment with a shorter delay, and more had received prior IVT, according to the univariate analysis. Most clinical comorbidities and stroke mechanisms were not significantly different between the two groups, as shown in Table 1.

**Figure 2:**
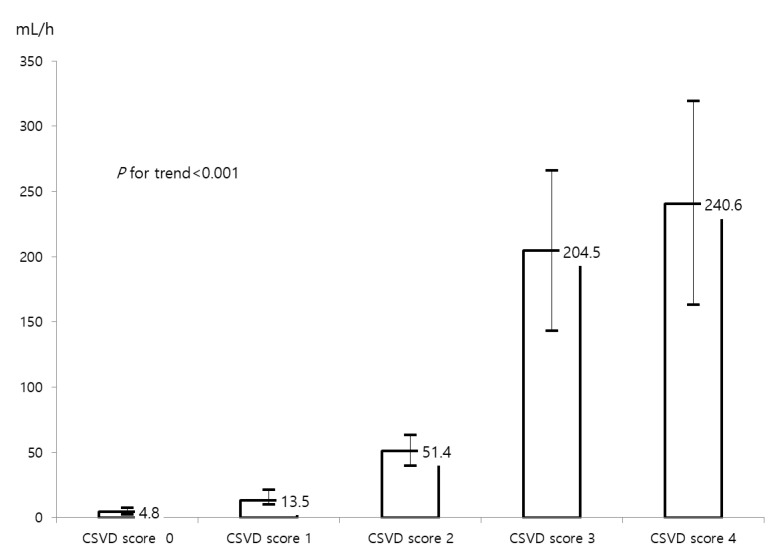
Box plot showing mean IGR according to CSVD score. Abbreviations: IGR, infarct growth rate; CSVD, cerebral small vessel disease.

**Figure 3:**
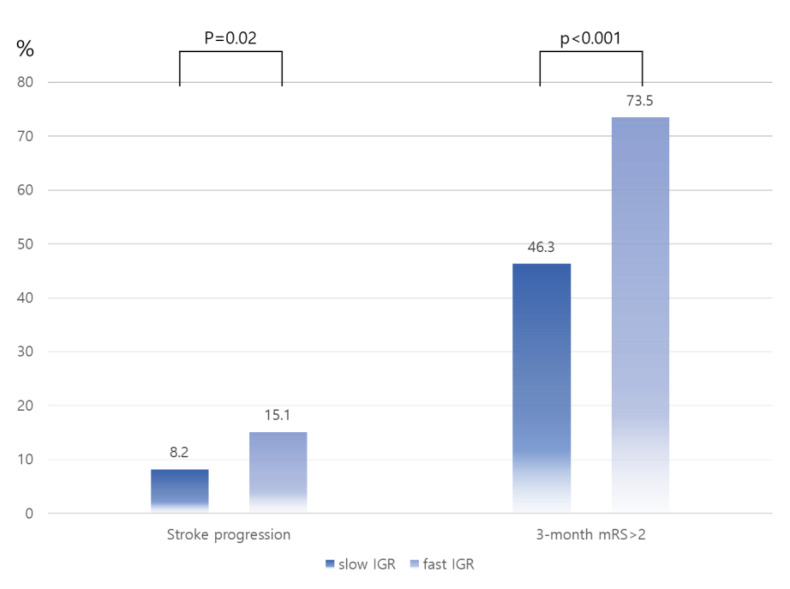
Distributions of stroke outcomes according to IGR. Abbreviations: IGR, infarct growth rate; mRS, modified Rankin Scale.

The fast IGR group had a higher proportion of high CSVD scores than the slow IGR group (24.4% vs. 0.8%, *p* < 0.001). The increase in cerebral small vessel disease (CSVD) score correlated with faster IGR (*p* for trend < 0.001, Figure 2). The group with fast IGR had a higher rate of stroke progression than the slow IGR group (15.1% vs. 8.2%, *p* = 0.02, Figure 3). Additionally, the fast IGR group had higher rates of poor functional outcomes at 3 months (mRS > 2) compared to the slow IGR group (73.5% vs. 46.3%, *p* < 0.001, Figure 3).

In multivariate analysis, a high CSVD score increased the risk of fast IGR after EVT (OR [95% CI], 26.26 [6.26–110.14], *p* < 0.001). High CSVD scores were significantly linked to increased stroke progression and poor functional outcome at 3 months, according to Table 2. Based on a sensitivity analysis, most CSVD markers (such as deep WMM Fazekas 2–3, periventricular WMH 3, EPVS, and CMB) were connected to fast IGR. However, lacunes were not correlated with fast IGR (Table 3).

## 4. Discussion

Our study included a large cohort of patients with anterior circulation stroke who underwent EVT, allowing a comprehensive analysis of the influence of CSVD on IGR and stroke outcomes. The main findings of this study indicate that a high CSVD burden, as reflected by our CSVD score, was significantly associated with faster IGR after EVT. In addition, patients in the fast IGR group had higher rates of stroke progression and poor functional outcomes at 3 months compared with those in the slow IGR group.

The observed relationship between CSVD burden and IGR suggests that CSVD may play a critical role in influencing the extent of ischemic injury in LVO stroke. Experimental and clinical studies have suggested that CSVD may affect the collateral circulation via vasodilatory responses, which could affect salvageable penumbral tissue in acute stroke [3]. Our study included only patients with good collateral status; we hypothesized that other pathophysiological mechanisms of CSVD burden may contribute to IGR. CSVD covers a range of pathological changes affecting the microvasculature of the brain, including arterioles, venules, and capillaries [23]. These pathological features may comprise vascular remodeling, endothelial dysfunction, and vessel wall thickening. These microvascular changes associated with CSVD may lead to impaired cerebral blood flow and altered perfusion [24]. CSVD has been associated with increased permeability of the BBB [25,26]. When the BBB is compromised, harmful substances, inflammatory molecules, and immune cells can more easily enter the brain. This, in turn, can trigger and worsen neuroinflammation and tissue damage, potentially contributing to the progression of ischemic injury [23,26]. Based on our findings, clinicians can use initial neuroimaging findings to identify markers of CSVD burden and select patients for EVT. However, further investigation is necessary to understand the precise mechanisms involved and to develop specific interventions that can minimize the impact of CSVD on ischemic injury. The results of our study have clinical significance in the context of EVT for LVO stroke. EVT is a major advance in acute stroke treatment, and its efficacy often depends on the concept of salvaging penumbral tissue while minimizing the expansion of the ischemic core. Patients with slower IGR are more likely to have a larger penumbral region, making them potential candidates for extended therapeutic windows, as demonstrated in studies such as DEFUSE 3. This suggests that identifying markers associated with accelerated infarct growth, such as CSVD burden, may help clinicians better stratify patients for EVT and predict their outcomes.

Interestingly, our study revealed that each CSVD marker was significantly linked with fast IGR in LVO patients. Extensive deep WMM, which reflects extensive microvascular damage, could decrease the integrity of white matter tracts and lower their resistance to ischemic insult [27]. Consequently, the ischemic injury may quickly propagate along compromised pathways, causing accelerated IGR. Severe periventricular white matter hyperintensities (WMHs) can disrupt normal fluid dynamics, impairing the brain’s ability to compensate for changes in blood flow [23,28]. This disruption may worsen the progression of ischemic injury by limiting fluid drainage and potentially causing edema in these regions. EPVS may disrupt the normal microenvironment around blood vessels. This could affect the delivery of oxygen and nutrients to the surrounding brain tissue, making it more susceptible to infarction [29]. Changes in the perivascular spaces may be associated with altered vasoreactivity, impacting the vessels’ ability to regulate blood flow. CMBs can occur in different regions of the brain and are linked with vascular fragility. The presence of CMBs indicates ongoing vascular pathology and fragility [30]. Additionally, these microbleeds can damage surrounding brain tissue and may contribute to a more rapid expansion of infarcted areas. The hemorrhagic nature of microbleeds can worsen local inflammation and tissue damage. While lacunes are linked to CSVD, they may not have the same direct impact on IGR as other CSVD markers in this study. This is because lacunes are usually clear, distinct infarcts, whereas other CSVD markers may affect a wider area of the brain through mechanisms like altered blood flow, perfusion, and microvascular dysfunction.

In addition, our study showed that a high CSVD burden not only increased the risk of rapid IGR, but also correlated with higher rates of stroke progression and poor functional outcomes at 3 months. The association between CSVD burden and stroke outcomes remains controversial [4,6,31]. Our findings highlight the need for a more comprehensive assessment of CSVD in stroke patients, as it could serve as a potential prognostic factor and guide treatment decisions. Future research may explore specific interventions or strategies to mitigate the impact of CSVD on stroke progression and improve outcomes in this patient population.

This study has several limitations. First, its retrospective nature restricts the ability to establish causal relationships, despite adjusting for several confounders. This study did not investigate the mechanistic pathways through which CSVD may influence infarct growth. Future research can explore these mechanisms in greater detail. Second, DWI was the primary method used to assess IGR in this study. DWI is a useful tool, but it has limitations in assessing the complete extent of ischemic injury since it only focuses on the acute phase. Combining DWI with other imaging techniques, like perfusion imaging, could lead to a more comprehensive understanding of IGR. Furthermore, the evaluation of CSVD burden relied on a CSVD score, which combines different neuroimaging features related to CSVD. The scoring system may not encompass all aspects of CSVD, and there may be an inherent interobserver variability in its computation. Nonetheless, our study maximized the generalizability by having two experienced neurologists conduct the MRI review with high reliability. Furthermore, our analysis focused on the baseline burden of CSVD and its correlation with IGR and stroke outcomes. Nevertheless, the longitudinal monitoring of CSVD progression and its possible influence on stroke outcomes were not included in this study. Long-term studies may provide further insights on this matter. Additionally, while this study adjusted for multiple confounding factors, there may be unmeasured or residual confounding variables that were not accounted for in the analysis, which could influence the observed associations. Unfortunately, vascular characteristics (tortuosity, elongation, and variants) of extracranial arteries were not available in this study. The severe stenosis of extracranial artery may increase the risk of CSVD [32,33]. Although other vascular characteristics of extracranial arteries were not available in this study, the stenosis of cervical ICA measured using NASCET criteria ≥50% was available. According to high CSVD, the proportion of ICA stenosis ≥50% was not significantly different in this study (no high CSVD vs. high CSVD: 41.0% vs. 36.4%, respectively, *p* = 0.77). Because the carotid stenosis was not different in both CSVD groups and we enrolled only the population with good collateral status before EVT, our result explaining the effect of CSVD on fast IGR might be reliable.

In conclusion, our study emphasizes the correlation between CSVD burden and infarct growth rate in LVO stroke patients undergoing EVT. Understanding the impact of cerebrovascular small vessel disease (CSVD) on stroke progression has the potential to improve patient selection for endovascular therapy (EVT) and enhance clinical outcomes. Further investigations are needed to explore the underlying mechanisms of this relationship and to develop targeted interventions that can mitigate the harmful effects of CSVD in acute ischemic stroke.

## Figures and Tables

**Table 1:** Baseline characteristics according to IGR.

	Slow IGR (n = 257)	Fast IGR (n = 238)	*p*-Value
Age, year (SD)	69.6 (13.6)	70.0 (12.3)	0.38
Male, n (%)	153 (59.5)	134 (56.3)	0.52
Initial NIHSS, score (IQR)	13 (9–18)	16 (13–18)	<0.001
Time from stroke onset to arrival, h (IQR)	2.7 (0.8–8.7)	1.1 (0.6–2.5)	<0.001
Time from stroke onset to imaging, h (IQR)	3.4 (1.3–7.0)	1.6 (1.0–2.9)	<0.001
Stroke subtypes, n (%)			0.07
LAA	66 (25.7)	50 (21.0)	
CE	138 (53.7)	152 (63.9)	
others	53 (20.6)	36 (15.1)	
Prior stroke, n (%)	54 (21.0)	51 (21.4)	0.91
Hypertension, n (%)	164 (63.8)	146 (61.3)	0.58
Diabetes mellitus, n (%)	70 (27.2)	60 (25.2)	0.61
Hyperlipidemia, n (%)	50 (19.5)	38 (16.0)	0.35
Current smoking, n (%)	43 (16.7)	32 (13.4)	0.32
Prior antithrombotics, n (%))	91 (35.4)	89 (37.4)	0.71
Prior statin, n (%)	50 (19.5)	42 (17.6)	0.65
Prior IVT, n (%)	105 (40.9)	143 (60.1)	<0.001
Occlusion site, n (%)			0.003
M1	192 (74.7)	203 (85.3)	
distal M1/M2	52 (20.2)	22 (9.2)	
ICA	13 (5.1)	13 (5.5)	
Successful reperfusion, n (%)	206 (80.2)	182 (76.5)	0.33
CSVD score, n (%)			<0.001
0	179 (69.6)	44 918.5)	
1	56 (21.8)	61 (25.6)	
2	22 (8.6)	76 (31.9)	
3	0 (0.0)	46 (19.3)	
4	0 (0.0)	11 (4.6)	

Abbreviation: IGR, infarct growth rate; SD, standard deviation; NIHSS, National Institute of Health Stroke Scale; IQR, interquartile range; LAA, large-artery atherosclerosis; CE, cardioembolism; IVT, intravenous thrombolysis; M1/M2, middle cerebral artery; ICA, internal carotid artery; CSVD, cerebral small vessel disease.

**Table 2:** Multivariate analysis showing effect of high CSVD on IGR and stroke outcomes in LVO stroke.

	Fast IGR		Stroke Progression		3-Month mRS > 2	
	OR	95%CI	OR	95%CI	OR	95%CI
High CSVD	26.26	6.26–110.14	5.83	2.75–12.36	4.07	1.85–8.95
Age	0.998	0.98–1.02	0.99	0.97–1.02	1.05	1.03–1.07
Male	0.97	0.63–1.49	0.45	0.23–0.87	0.74	0.48–1.16
Time from stroke onset to imaging time	0.68	0.27–1.70	0.88	0.23–3.45	0.56	0.22–1.46
Time from stroke onset to arrival time	1.29	0.52–3.24	1.15	0.30–4.50	1.85	0.72–4.80
Initial NIHSS	1.05	1.01–1.08	0.94	0.89–0.996	1.11	1.07–1.16
Stroke subtypes						
Others	reference		reference		reference	
LAA	1.24	0.65–2.36	1.76	0.64–4.86	1.13	0.59–2.18
CE	1.48	0.83–2.63	1.82	0.71–4.66	0.73	0.41–1.30
Prior IVT	1.41	0.87–2.27	0.76	0.39–1.51	0.83	0.52–1.35
Occlusion site	0.70	0.47–1.05	1.63	1.04–2.58	1.16	0.78–1.72
Successful reperfusion	0.79	0.48–1.30	0.30	0.16–0.56	0.25	0.14–0.44

Abbreviation: CSVD, cerebral small vessel disease; IGR, infarct growth rate; LVO, large vessel occlusion; mRS, modified Rankin Scale; OR, odds ratio; CI, confidence interval; NIHSS, National Institute of Health Stroke Scale; LAA, large artery atherosclerosis; CE, cardioembolism; IVT, intravenous thrombolysis.

**Table 3:** Multivariate analysis showing effect of each CSVD marker on fast IGR.

	OR	95% CI	*p*-Value
Age	1.004	0.99–1.02	0.65
Male	0.65	0.59–1.52	0.82
Time from stroke onset to imaging time	0.60	0.22–1.66	0.33
Time from stroke onset to arrival time	1.52	0.55–4.21	0.42
Initial NIHSS	1.01	0.97–1.05	0.75
Stroke subtypes	0.93	0.66–1.32	0.69
Prior IVT	1.42	0.83–2.41	0.20
Occlusion site	0.70	0.45–1.10	0.12
CSVD markers			
DWMH Fazekas 2–3	3.24	2.05–5.13	<0.001
PWMH Fazekas 3	1.15	1.08–1.23	<0.001
EPVS (Score 2–4)	3.47	1.21–9.94	0.02
CMB(s) ≥ 1	13.74	3.14–60.15	0.001
Lacune(s) ≥ 1	1.54	0.88–2.67	0.13

Abbreviation: CSVD, cerebral small vessel disease; IGR, infarct growth rate; OR, odds ratio; CI, confidence interval; NIHSS, National Institute of Health Stroke Scale; IVT, intravenous thrombolysis; DWMH, deep white matter hyperintensity; PWMH, periventricular white matter hyperintensity; EPVS, enlarged perivascular space; CMB, cerebral microbleed.

## Data Availability

The data that support the findings of this study are available upon reasonable request from the corresponding author.

## Informed Consents Statement

All patients signed a written informed consent to participate in the study.
